# Mycotoxin occurrence and risk assessment in plant-based meat, cheese, and fish alternatives based on an adapted UHPLC-MS/MS multi-method

**DOI:** 10.1007/s12550-026-00636-2

**Published:** 2026-02-03

**Authors:** Sarah Schneidemann-Bostelmann, Yannick Otting, Fabian Dick, Thijs Lefever, Stefan Asam, Michael Rychlik

**Affiliations:** Chair of Analytical Food Chemistry, Technical University of Munich, Freising, Germany

**Keywords:** Multi mycotoxins, Aflatoxins, *Fusarium* toxins, *Alternaria* toxins, Plant-based alternatives, SIDA, QuEChERS, LC-MS/MS

## Abstract

Plant-based alternatives to animal-derived products have gained significant prominence in recent years, with the market for these products experiencing continuous growth. In response to this trend, we developed an ultra-high-performance liquid chromatography method, coupled with tandem mass spectrometry (UHPLC-MS/MS) for the simultaneous quantification of 15 mycotoxins from *Fusarium* (deoxynivalenol (DON), DON-3-glucoside (D3G), 3-acetyl-DON, 15-acetyl-DON, HT-2 toxin, T-2 toxin), *Alternaria* (alternariol, alternariolmonomethylether (AME), tenuazonic acid (TeA)), and *Aspergillus* species (aflatoxin (AF) B1, B2, G1, G2, sterigmatocystin (STC), ochratoxin A) in plant-based meat, cheese, and fish substitutes. Sample extraction was performed using a QuEChERS (quick, easy, cheap, effective, rugged, and safe) approach, followed by dispersive solid phase extraction (dSPE) for clean-up. Quantification was achieved through stable isotope dilution analysis (SIDA). The method demonstrated limits of detection (LOD) ranging from 0.01 µg/kg (AFB2 and STC) to 14.1 µg/kg (D3G) and limits of quantification (LOQ) between 0.02 µg/kg (STC) and 41.9 µg/kg (D3G). This method was successfully applied to quantify mycotoxins in 32 plant-based alternative products, revealing contamination predominantly with *Alternaria* toxins such as AME and TeA. Seitan-based products were identified as having the highest levels of contamination. In total, 27 out of 32 analyzed products contained at least one of the target mycotoxins. With the obtained data, we performed a risk assessment and revealed a potential risk, especially for toddlers, but also for individuals of all ages, due to the presence of *Alternaria* toxins. However, none of the detected concentrations exceeded the established regulatory limits or recommended values, as plant-based alternatives are not specifically included in the relevant food safety regulations.

**Supplementary information:**

The online version contains supplementary material available at 10.1007/s12550-026-00636-2.

## Introduction

In recent years, the rapid growth of the global population and the depletion of natural resources have emerged as major challenges worldwide. Consequently, there is a growing demand for sustainable, nutritious, and health-conscious food options. It is well-established that meat production significantly impacts environmental concerns, including greenhouse gas emissions and excessive water consumption (Quintieri et al. 2023). This environmental impact is one of the key factors contributing to the growing importance of plant-based foods, such as plant-based meat alternatives (PBMAs). In addition to environmental considerations, other driving factors include ethical concerns, particularly regarding animal welfare, and potential health benefits. Plant-based diets have been reported to positively impact human health by lowering blood pressure and reducing the risk of cardiovascular diseases (Boukid 2021; Mihalache et al. 2022; Elhalis et al. 2023).

A new wave of plant-based products designed to replicate the characteristics of real meat is already available on the market. These PBMAs consist of plant proteins that are carefully structured and formulated to resemble meat in terms of flavor, appearance, texture, and nutritional composition (Joshi and Kumar 2015; Kumar et al. 2017; Elhalis et al. 2023). The importance of the PBMA market is demonstrated by its remarkable growth rate of 14.7%, increasing from 7.9 billion USD in 2022 to an estimated value of 15.7 billion USD by 2027 (Markets and Markets 2022). Focusing on Germany, the per capita consumption of PBMAs is expected to reach 0.76 kg per person in 2028, compared to just 0.14 kg in 2018 and 0.44 kg in 2022 (Statista 2023). The significant role of PBMAs in the European alternative protein products market is also evidenced by the proportion of product launches incorporating alternative proteins in Europe in 2023, where PBMAs accounted for 58% of the total. In contrast, other product categories, such as alternative dairy products, represented only 0.87% (Innova Market Insights 2024). Nonetheless, the market for these other alternative product categories is also experiencing growth. For instance, the market for milk alternatives in Switzerland expanded from 56.4 million Swiss francs (SFr) in 2021 to 69 million SFr in 2023. Looking closely at alternative cheese products, the market for cheese alternatives increased from 11.8 million SFr in 2021 to 13.4 million SFr in 2023 (COOP Marktforschung 2022, 2024).

This study focuses on PBMAs, plant-based cheese alternatives (PBCAs), and plant-based fish alternatives (PBFAs). The mentioned plant-based alternative products (PBAPs) are frequently derived from proteins found in legumes, such as soybeans, peas, chickpeas, and lentils, but can also be based on wheat gluten (then named seitan) (Schreuders et al. 2021; Mihalache et al. 2022; Quintieri et al. 2023). Especially products based on pea proteins are gaining increasing attention due to their functional properties, nutritional benefits, and low allergenic potential (Osen et al. 2014, 2015; Mihalache et al. 2022). Seitan, composed primarily of pure gluten, is widely consumed in vegetarian and vegan diets. Traditionally used in East Asian cuisine, wheat gluten has gained popularity in Western markets over the past decades. This versatile ingredient is commonly found in various meat substitute products, including burgers, sausages, and nuggets, where it is often seasoned and colored to enhance its resemblance to conventional meat. As one of the most widely available PBMA, seitan plays a significant role in the growing market for sustainable and ethical protein sources (Mihalache et al. 2022; Quintieri et al. 2023; Malav et al. 2015; Schreuders et al. 2021). However, very little information is available regarding its contamination with natural toxins, of which the mycotoxins are of outstanding importance. Mihalache et al. (2022) already mentioned this lack of knowledge. The presence of mycotoxins in PBMAs has been well documented, particularly in soy-based products (Schollenberger et al. 2007; Rodríguez-Carrasco et al. 2019; Oleynikova et al. 2020; Tian et al. 2022; Mihalache et al. 2022). However, there is limited data available on mycotoxin contamination in PBMAs derived from other plant protein sources, such as pea and wheat (Mihalache et al. 2023, 2024).

In general, mycotoxins can be defined as: *“[…] secondary fungal metabolites*,* toxic to humans and animals. Toxigenic fungi often grow on edible plants*,* thus contaminating food and feed.”* (Berthiller et al. 2013). *These* toxins are predominantly produced by molds belonging to the genera *Aspergillus*,* Penicillium*,* Fusarium*, and *Alternaria*. They can have harmful effects, including cytotoxicity, immune system modulation, genotoxicity, and carcinogenicity, posing a significant health risk to humans (WHO 2023). It has already been reported that vegetarians may have a higher exposure to ochratoxin A (OTA) than omnivore-living people, which is proven by the fact that more OTA was found in their urine than in the urine of meat-eating people (Penczynski et al. 2022).

This is why this study aimed to determine and quantify 15 mycotoxins, including type A and B trichothecenes (deoxynivalenol (DON), DON-3-glucoside (D3G), 3-acetyl-DON (3-AcDON), 15-acetyl-DON (15-AcDON), HT-2 toxin (HT-2), T-2 toxin (T-2), aflatoxins (AFs) G1, G2, B1, B2, and sterigmatocystin (STC), *Alternaria* toxins (alternariol (AOH), alternariolmonomethylether (AME) and tenuazonic acid (TeA) and OTA in 32 different PBMAs, PBCAs, and PBFAs bought in German and Belgian supermarkets. The focus was on products containing various protein types other than soy. We used an ultra-high-performance liquid chromatography method coupled with tandem mass spectrometry (UHPLC-MS/MS) following clean-up by QuEChERs (quick, easy, cheap, effective, rugged, and safe) and dispersive solid phase extraction (dSPE). This method was initially developed by Dick et al. (2024) for cereals and was customized for fatty matrices. The range of analytes was also adjusted for the respective matrices; some analytes were omitted, and AFs, STC, and OTA were added to the method, which was validated according to Vogelgesang and Hädrich (1998). Since our method includes the determination of TeA, we decided to continue using the method of Dick et al. (2024) rather than performing a simple extraction followed by LC-MS/MS analysis as described by Mihalache et al. (2024).

## Materials and methods

### Chemicals and reagents

Acetonitrile (ACN) (LC-MS grade) and methanol (MeOH) (LC-MS grade) were obtained from Honeywell Riedel-de Haën (Seelze, Germany). Ultrapure water (H₂O) (LC-MS grade) was purchased from Th. Geyer (Renningen, Germany). Sodium chloride (NaCl) in analytical grade was supplied by VWR (Ismaning, Germany). Anhydrous magnesium sulfate (anhydrous MgSO₄), formic acid (FA), ammonia solution (NH₄OH), and ammonium formate solution (NH_4_HCO_2_) were acquired from Sigma Aldrich (Steinheim, Germany) in analytical grade or higher as well as the dSPE tubes Supel™ QuE PSA/C18 tube (400 mg Discovery™ DSC-18, 1200 mg anhydrous MgSO_4_, 400 mg Supelclean™ PSA (primary secondary amine)).

### Analytical standards

Following reference standards and isotope-labeled internal standards were purchased from sources listed in brackets: aflatoxins in ACN (containing AFG1, AFG2, AFB1, and AFB2; 1 µg/ml each), D3G, [^13^C_21_]-D3G, [^13^C_17_]−3-AcDON, [^13^C_22_]-HT-2 (Romer Labs, former Biopure, Tulln, Austria), [^13^C_17_]**-**AFG1, [^13^C_17_]-AFG2, [^13^C_17_]-AFB1, [^13^C_17_]-AFB2, [^13^C_18_]-STC, [^13^C_20_]-OTA and [^13^C_15_]-DON (Fianovis, former Libios, Vindry-sur-Turdine, France), STC, DON, 3-AcDON and 15-AcDON (Romer Labs, former Coring System Diagnostix, Gernsheim, Germany), T-2 toxin (LGC Standards/Dr. Ehrenstorfer, Wesel, Germany), HT-2 toxin, OTA (Sigma Aldrich, Steinheim, Germany) and TeA (Merck, Darmstadt, Germany).

The referenced compounds AME and AOH were isolated from fungal extracts (Scheibenzuber et al. 2022), and the isotope-labeled internal standards [^13^C_4_]-T-2, [d_4_]-AOH, [d_4_]-AME, and [^13^C_6_^15^N]-TeA were synthesized at our chair as described previously in the literature (Asam and Rychlik 2006; Asam et al. 2009, 2011).

### Certified reference material

Certified reference material BCR™ – 263R (AFB1, AFB2, and AFG1 in defatted peanut meal) was obtained from LGC Standards (Wesel, Germany).

### Samples

Cashew kernels and all analyzed products were purchased in German and Belgian supermarkets.

### Preparation of stock solutions

Stock solutions of labeled and unlabeled toxins were prepared in ACN at a concentration of 10 µg/mL in general (except for AFs) and diluted to final concentrations of 1, 0.1, 0.01, and 0.001 µg/mL with ACN. The stock solutions were stored at − 28 °C.

### Grinding and freeze-drying

A representative amount of the sample (around 100 g) was freeze-dried for at least 48 h (Alpha 1–2 LD plus, Martin Christ Gefriertrocknungsanlagen GmbH, Osterode am Harz, Germany) and was then homogenized with a laboratory mill (Grindomix GM 200, Retsch GmbH, Haan, Germany). The samples were stored at − 28 °C until sample preparation.

### Sample preparation

The sample preparation was performed according to the protocol of Dick et al. (2024), with minor modifications.

One gram of the homogenized and freeze-dried sample was weighed into a 15 mL centrifuge tube. The sample was spiked with isotope-labeled internal standards, and the solvent was evaporated overnight under a fume hood. Following the spiking step, the sample underwent a three-step extraction process. Initially, 10 mL of ACN/H₂O (80/20, v/v) and 100 µL of FA were added, and the mixture was shaken on a horizontal shaker (Kombischüttler KL 2, Edmund Bühler GmbH, Hechingen, Germany) at 400 rpm for 60 min.

Subsequently, centrifugation was performed at 3,220 g for 5 min (Centrifuge 5810 R, Eppendorf AG, Hamburg, Germany), and the supernatant was collected in a 50 mL centrifuge tube. The residue was then re-extracted, first with 5 mL of ACN/H₂O (80/20, v/v) and 50 µL of FA for 30 min, followed by another round of centrifugation (3,220 g, 5 min). Finally, the residue was extracted once more with 5 mL of ACN/H₂O (70/30, v/v) and 50 µL of FA for 30 min, followed by centrifugation (3,220 g, 5 min).

After each extraction, the supernatants were combined in the same 50 mL centrifuge tube and subsequently acidified with 200 µL of FA. The supernatants were added to 1.8 g of anhydrous MgSO₄ and 0.45 g of NaCl, and the mixture was vortexed for 1 min as part of a QuEChERS cleanup procedure. After centrifugation (3,220 g, 5 min), 8 mL of the ACN phase was transferred to a d-SPE tube (Supel™ QuE PSA/C18 tube containing 400 mg Discovery™ DSC-18, 1.2 g anhydrous MgSO₄, and 400 mg Supelclean™ PSA). The mixture was shaken on a horizontal shaker for 15 min and centrifuged again (3,220 g, 5 min). The supernatant was then transferred to a 4 mL glass vial and evaporated to dryness under a nitrogen stream at 40 °C. This was done in two steps: initially, 4 mL of the supernatant was transferred to the vial and dried, and once the vial was nearly empty, the remaining supernatant was added and dried.

The dry residue was then reconstituted in 200 µL of MeOH/H₂O (60/40, v/v), and the solution was frozen for at least 30 min at − 28 °C. After membrane filtration (PVDF, 0.2 μm) into a 1.5 mL plastic tube, a 15-minute centrifugation was performed at 13,201 g and 4 °C (Laborzentrifuge 2K15, Sigma, Osterode am Harz, Germany). The supernatant was finally transferred into a 1.5 mL glass vial with a micro-insert, and the samples were stored at − 28 °C until further analysis.

### UHPLC-MS/MS analysis

For the UHPLC-MS/MS analysis, we took the previously established method according to Dick et al. (2024) and included the *Aspergillus* toxins AFB1, AFB2, AFG1, AFG2, OTA, and STC. However, some mycotoxins were omitted from the original method because they were not relevant for the kind of samples in this study.

The chromatographic separation was done on a Shimadzu Nexera X2 UHPLC system (Shimadzu, Kyoto, Japan). A Waters BEH C18 UHPLC column (Acquity BEH C18, 100 mm, 1.7 μm × 2.1 mm; Waters GmbH, Eschborn, Germany) was used, which was kept at 40 °C. The flow rate was 0.3 mL/min. The binary gradient system consisted of (A) 5 mM NH_4_HCO_2_ solution in H_2_O at pH 9 and (B) MeOH. For solvent A, the pH was adjusted using a 25% NH₄OH solution. The binary gradient started at 5% B and was held for two minutes. Then, B was raised to 18% in 1 min, followed by a gradual increase to 25% of solvent B over the next 2 min. Subsequently, the concentration of B was ramped up to 90% over 8 min, then further elevated to 99% within 0.5 min, where it was maintained for 2 min. The gradient then returned to 5% B over 3.5 min, with a 5-minute equilibration phase at the end. An injection volume of 10 µL was selected, and to improve peak shape, 40 µL of 5 mM NH_4_HCO_2_ solution in H_2_O (pH 9) was co-injected. In this process, 2 times 20 µL were injected, each before and after the 10 µL sample injection.

The UHPLC system was connected to a Shimadzu 8050 triple quadrupole mass spectrometer (Shimadzu Corporation, Kyoto, Japan). The ion source parameters were set to the following conditions: interface temperature 350 °C, heat block temperature 450 °C, desolvation temperature 150 °C, interface voltage 3 kV for positive ionization and − 3 kV for negative ionization, heating gas flow 10 L/min, drying gas flow 10 L/min, nebulizing gas flow 3 L/min and collision-induced dissociation gas pressure 270 kPa. Positive and negative electrospray ionization mode within a single LC-MS/MS run was enabled using polarity switching. All measurements were performed in multiple reaction monitoring (MRM) mode. The mass transitions of all analytes are shown in Table 1.

**Table 1:** LC-MS/MS parameters of the analyzed mycotoxins

Analyte	Measured ion	Precursor ion (m/z)	Product ions (m/z)	Q1 Pre bias (V)	Collision energy [V]	Q3 Pre bias (V)	Retention time (min)
			Qualifier/Quantifier				
DON	[M + H]^+^	297.15	249.20/231.15	−14.0/−18.0	−11.0/−12.0	−18.0/−26.0	5.5
[^13^C_15_]-DON	[M + H]^+^	312.15	263.20/245.15	−14.0/−18.0	−11.0/−12.0	−18.0/−26.0	5.5
D3G	[M - H]^−^	457.15	427.20/247.10	12.0/12.0	19.0/20.0	28.0/24.0	5.8
[^13^C_21_]-D3G	[M - H]^−^	478.30	447.05/261.30	22.0/12.0	17.0/20.0	44.0/24.0	5.8
3-AcDON	[M + H]^+^	339.10	231.25/175.20	−16.0/−16.0	−13.0/−25.0	−26.0/−20.0	8.3
[^13^C_15_]−3-AcDON	[M + H]^+^	356.10	245.25/186.20	−16.0/−16.0	−13.0/−25.0	−26.0/−20.0	8.3
15-AcDON	[M + H]^+^	339.30	279.20/261.20	−16.0/−12.0	−13.0/−12.0	−30.0/−20.0	8.4
HT-2	[M + NH_4_]^+^	442.20	263.25/215.10	−22.0/−22.0	−14.0/−14.0	−10.0/−24.0	11.1
[^13^C_22_]-HT-2	[M + NH_4_]^+^	464.20	278.25/200.20	−22.0/−22.0	−14.0/−17.0	−10.0/−44.0	11.1
T-2	[M + NH_4_]^+^	484.15	185.20/215.25	−26.0/−26.0	−20.0/−19.0	−20.0/−24.0	11.6
[^13^C_4_]-T-2	[M + NH_4_]^+^	488.15	307.15/216.25	−26.0/−26.0	−12.0/−19.0	−16.0/−24.0	11.6
AFG2	[M + H]^+^	331.20	189.10/115.20	−18.0/−14.0	−42.0/−65.0	−20.0/−24.0	8.9
[^13^C_17_]- AFG2	[M + H]^+^	348.20	199.10/123.20	−18.0/−14.0	−42.0/−65.0	−20.0/−24.0	8.9
AFG1	[M + H]^+^	329.00	243.15/200.10	−16.0/−14.0	−26.0/−43.0	−26.0/−22.0	9.2
[^13^C_17_]- AFG1	[M + H]^+^	346.00	257.15/212.10	−16.0/−14.0	−26.0/−43.0	−26.0/−22.0	9.2
AFB2	[M + H]^+^	315.00	287.15/259.10	−16.0/−16.0	−26.0/−30.0	−20.0/−18.0	9.6
[^13^C_17_]- AFB2	[M + H]^+^	332.00	303.15/274.10	−16.0/−16.0	−26.0/−30.0	−20.0/−18.0	9.6
AFB1	[M + H]^+^	313.00	285.15/241.10	−16.0/−16.0	−23.0/−36.0	−20.0/−26.0	9.8
[^13^C_17_]- AFB1	[M + H]^+^	330.00	301.15/256.10	−16.0/−16.0	−23.0/−36.0	−20.0/−26.0	9.8
STC	[M + H]^+^	324.90	310.20/281.15	−16.0/−16.0	−23.0/−36.0	−20.0/−26.0	12.3
[^13^C_18_]-STC	[M + H]^+^	342.90	327.20/298.15	−16.0/−16.0	−23.0/−36.0	−20.0/−26.0	12.3
OTA	[M - H]^−^	402.30	358.20/167.10	20.0/20.0	21.0/36.0	14.0/14.0	10.0
[^13^C_20_]-OTA	[M - H]^−^	422.30	377.20/174.10	20.0/20.0	21.0/36.0	14.0/14.0	10.0
TeA	[M - H]^−^	196.40	111.95/139.00	22.0/22.0	19.0/25.0	26.0/34.0	4.5
[^13^C_6_^15^N]-TeA	[M - H]^−^	203.40	112.95/142.00	22.0/22.0	25.0/19.0	34.0/26.0	4.5
AOH	[M - H]^−^	257.30	213.00/214.85	28.0/26.0	25.0/23.0	34.0/14.0	9.2
[d_4_]-AOH	[M - H]^−^	261.30	217.00/218.85	28.0/26.0	25.0/23.0	34.0/14.0	9.2
AME	[M - H]^−^	271.10	256.10/255.10	20.0/20.0	23.0/31.0	24.0/24.0	12.3
[d_4_]-AME	[M - H]^−^	275.10	260.10/259.10	20.0/20.0	23.0/31.0	24.0/24.0	12.3

### Calibration and quantitation

All toxins were quantified using stable isotope dilution analysis (SIDA). For recording the calibration curves, stable isotope-labeled standards (S) were kept at a constant amount and mixed with varying quantities of their non-labeled counterparts (A) to achieve molar ratios of n(A)/n(S) ranging from 0.01 to 100 (1:100, 1:50, 1:10, 1:5, 1:2, 1:1, 2:1, 5:1, 10:1, 50:1, 100:1). By plotting the ratio of peak areas [A(A)/A(S)] against the corresponding molar ratios [n(A)/n(S)], response curves were generated using linear regression. Their linearity was assessed according to the Mandel fitting test (Mandel 1964). 15-AcDON was quantified using response curves with [^13^C_15_]−3-AcDON as a structurally similar internal standard.

### Method validation

#### Search for a toxin-free blank matrix

Various nuts and oilseeds were tested for their mycotoxin content, but none were completely devoid of the analytes under study. Finally, a sample of cashew kernels (purchased in a German supermarket) was identified that contained only trace amounts of DON and AME. After homogenization, cashew kernels were used as a blank matrix for the validation of the mycotoxins D3G, 3-AcDON, 15-AcDON, T-2, HT-2, AFB1, AFB2, AFG1, AFG2, STC, OTA, TeA, and AOH. Since no natural matrix completely free of DON and AME was found, a mixture of mycotoxin-free potato starch (Merck KGaA, Darmstadt, Germany) and mycotoxin-free coconut fat (purchased in a German supermarket) in the ratio 50/50 (w/w) was used for the validation of these two toxins.

#### LODs and LOQs

The limits of detection (LODs) and limits of quantification (LOQs) were determined according to Vogelgesang and Hädrich (1998). Therefore, the blank matrix was spiked in triplicate with the unlabeled analytes in four different concentrations and their respective internal standard (for details, please refer to Supplementary Table 9 in the SI). The samples were then processed according to the described sample preparation procedure.

#### Recovery

Blank material was spiked with four concentrations of each unlabeled analyte and their corresponding internal standards, both diluted in ACN (for details, please refer to Supplementary Table 8 in the SI). After the solvent had evaporated at room temperature, the samples were analyzed using the previously described workup and LC-MS/MS procedure. Recovery rates were calculated as the ratio between the measured toxin concentrations and the spiked amounts times 100.

#### Precision

The blank matrix was spiked in triplicate with all unlabeled analytes and corresponding internal standards (for details, please refer to Supplementary Table 10 in the SI). The spiked matrix was processed according to the described sample preparation procedure, followed by LC-MS/MS analysis. To determine the intra-day precision, the same sample was worked up three times on the same day (*n* = 3), and the inter-day precision was determined by working up three samples on three different days (*n* = 9). To evaluate instrument precision, a solution containing all unlabeled analytes and their corresponding internal standards, dissolved in ACN, was injected into the LC-MS/MS system 10 consecutive times (*n* = 10).

#### Trueness

A certified reference material (defatted peanut meal) containing AFB1 (17.1 ± 2.4 µg/kg), AFB2 (3.0 ± 0.4 µg/kg), and AFG1 (3.0 ± 0.5 µg/kg) was worked up and analyzed as described previously to test the trueness of the determination.

### Analysis of mycotoxins in PBAPs

A total of 32 samples were purchased from Belgian and German supermarkets and included 26 PBMAs, 4 PBCAs, and two PBFAs made from plant proteins derived from various sources. The analysis covered 13 grain- or cereal-based products (including nine seitan products), nine products with legume protein, nine products based on nuts and oilseeds, and one product containing both cereal and legume protein. The quantification of the 15 mycotoxins in commercial samples was performed in triplicate.

### Data analysis

For the peak integration, the software LabSolutions version 5.118 (Shimadzu, Kyoto, Japan) was used. Response curves, Mandel fitting tests, and analyte concentrations were calculated using Microsoft Excel for Mac, Version 16.92 (Microsoft Co, Redmond, WA, USA). Quantitative data were visualized using OriginLab 2024b (OriginLab Corporation, Northampton, MA, USA).

### Risk evaluation

#### Consumption data

To conduct a risk assessment, it was first necessary to determine the daily consumption of PBAPs. For this purpose, the German national consumption survey NVS II (Heuer et al. 2015) was used for adults, adolescents, and elderly people, while the KiESEL study (Spiegler et al. 2024) was used for children (toddlers and preschoolers). Unfortunately, these studies do not provide data on vegetarian and vegan alternative products. For the risk assessment of the 26 PBMAs analyzed in this study, we assumed that PBMAs entirely replaced meat consumption in the diet. The consumption of fish and cheese was disregarded, as only 6 out of the 32 analyzed samples served as substitutes for these product groups. Since the consumption data in the mentioned studies is divided by gender, we first calculated the average gender-independent consumption of meat and meat products within the specified age groups. This gives us the following consumption data for meat, meat products, and sausages: toddlers (1–3 years): 34 g/day; preschoolers (4–5 years): 53.5 g/day; adolescents (14–17 years): 118 g/day; adults (18–64 years): 116 g/day and elderly people (65–80 years): 84.5 g/day.

#### Handling of left-censored data

Upon calculating the contamination data, we encountered the problem of some measurement data falling below the LOQ, while many are falling below the LOD. Handling these left-censored data is crucial in risk assessment when converting mycotoxin contamination into exposure data. Therefore, we decided to follow the recommendation of the EFSA (2010) and apply the lower bound (LB), middle bound (MB), and upper bound (UB) approaches to process these measurements. For the LB calculation, all values below the LOQ are considered as 0. In the MB approach, values below the LOD are replaced with 1/2 LOD, while values greater than LOD but below LOQ are replaced with 1/2 LOQ. For the UB calculation, values below the LOD are replaced with the corresponding LOD value, and values greater than LOD but below LOQ are replaced with the corresponding LOQ value.

#### Calculation of estimated daily intake

Based on the consumption and contamination data, we calculated the estimated daily intake (EDI) as follows:

$$EDI\left[\mu g/\left(kg\;bw\cdot d\right)\right]=contimanation\left[\mu/kg\right]\cdot consumption\left[kg/kg\;bw\right]$$

For body weight (bw), we followed the recommendations according to EFSA (2012) (toddlers = 12.0 kg, preschoolers = 23.1 kg, adolescents = 61.3 kg, adults = 70.0 kg, and elderly people = 70.0 kg).

#### Health-based guidance values

For health-based guidance values (HBGV), we referred to the tolerable daily intake (TDI) set by EFSA, including 1 µg/kg bw per day for DON (group TDI for DON, 3-AcDON, 15-AcDON, and D3G) (EFSA 2017b; European Commission 2024a) and 0.02 µg/kg bw per day for the sum of T-2 and HT-2 (EFSA 2017a; European Commission 2024b).

No TDIs have been established for *Alternaria* toxins so far. Therefore, we used the threshold of toxicological concern (TTC) as HBGV for risk assessment. The TTC values are 1.5 µg/kg bw per day for TeA and 2.5 ng/kg bw per day for AOH and AME (EFSA 2016).

#### Calculation of hazard quotients

Then, we calculated the hazard quotient (HQ) by dividing the EDI by the determined HBGV, such as TDI or TTC, as follows:

$$\:HQ=EDI/HBGV$$

#### Calculation of margin of exposure

For AFB1 and OTA, the margin of exposure (MoE) was calculated as follows:

$$\:MoE={BMDL}_{10}/EDI$$

with BMDL_10_ being 0.4 µg/kg bw day for AFB1 (EFSA 2020a) and BMDL_10_ being 4.73 µg/kg bw day (due to non-neoplastic effects) and 14.5 µg/kg bw day (due to carcinogenicity) for OTA (EFSA 2020b).

## Results and discussion

### Sample preparation and LC-MS/MS

**Fig. 1:**
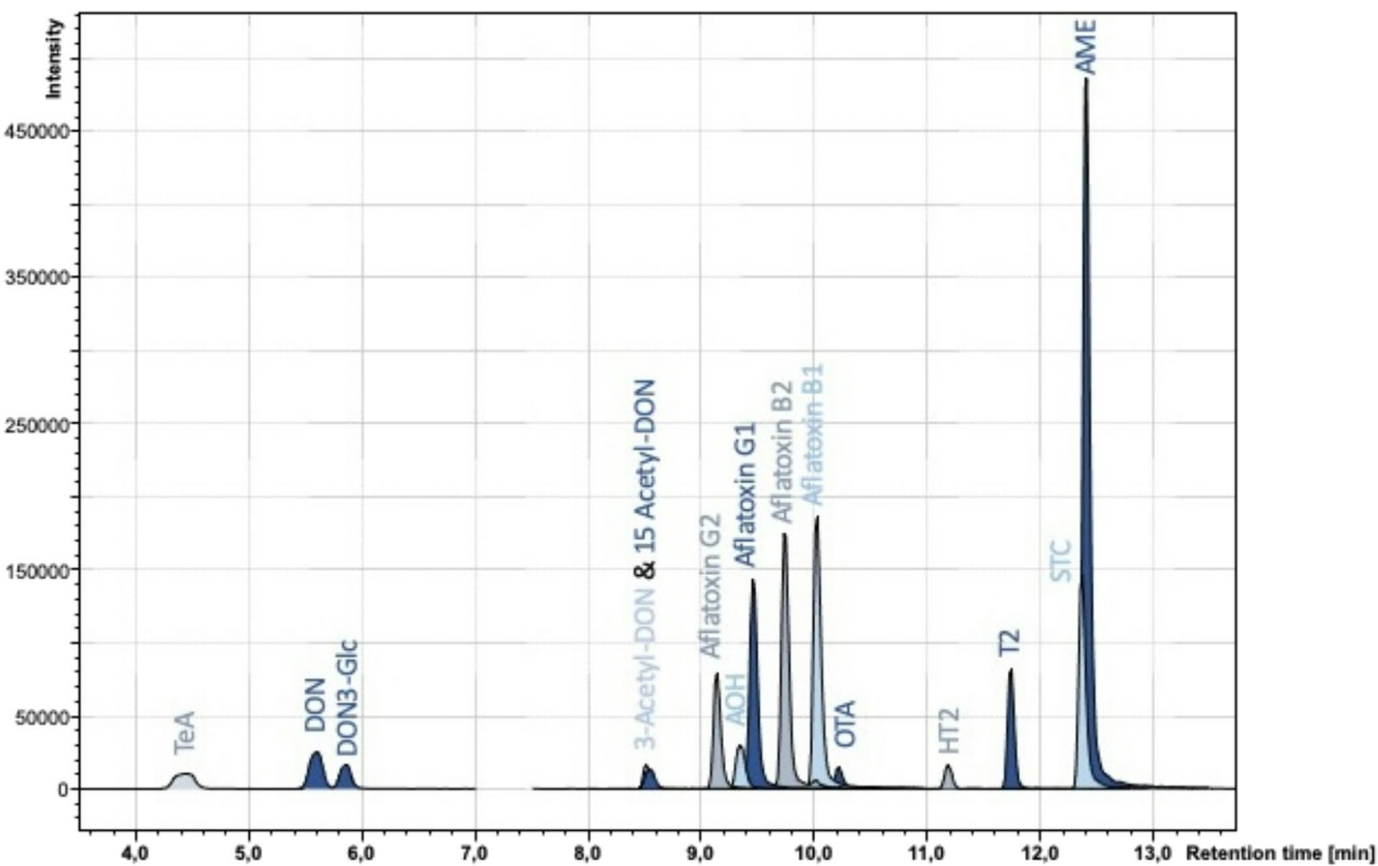
UHPLC-MS/MS chromatogram of all 15 analytes

A QuEChERS-based UHPLC-LS-MS/MS method was successfully developed and validated to simultaneously analyze 15 different toxins from *Alternaria*, *Aspergillus*, and *Fusarium* in fatty matrices. This method builds upon our previously published method for analyzing 24 different *Alternaria* and *Fusarium* toxins in cereals and cereal-based products (Dick et al. 2024). The method was expanded to include *Aspergillus* toxins such as OTA, STC, AFB1, AFB2, AFG1, and AFG2. However, we omitted some emerging and modified mycotoxins from the original method since we have decided to analyze only the mycotoxins that are regulated in the Commission Regulation 2023/915 for fatty matrices (European Commission 2023) or for which legal regulation is expected in the future (European Commission 2022). A chromatogram showing all mycotoxins is presented in Fig. 1. The sample preparation procedure involving triple extraction followed by QuEChERS and dSPE was chosen to maximize sensitivity and ensure effective cleanup in complex matrices.

### Calibration and quantitation

All analytes were quantified using SIDA with their corresponding isotope-labelled internal standard. Only 15-AcDON was analyzed using [^13^C_17_]−3-AcDON due to structural similarity. Linear regression was used to generate response curves, and their linearity was verified using Mandel’s fitting test (Mandel 1964). The calibration curves showed linearity within the molar ratios as follows: DON, AFB1, AFG1, AFB2, STC, T-2, HT-2, and AOH: 0.01–100; 3-AcDON, 15-AcDON, TeA, and AME: 0.01–50; AFG2: 0.01–10; D3G and OTA: 0.02–100.

### Validation

The search for a suitable blank matrix proved to be a challenge. We decided to use homogenized cashew kernels to validate 3-AcDON, 15-AcDON, D3G, T-2, HT-2, TeA, AOH, AFB1, AFB2, AFG1, AFG2, STC and OTA. Since we could not find a natural matrix free of DON and AME, we decided to use mycotoxin-free potato starch and mycotoxin-free coconut fat in the ratio 50/50 (w/w) as a blank matrix for the validation of these two toxins. A detailed summary of the validation is presented in Table 2.

**Table 2:** Validation data: limits of detection (LOD), limits of quantification (LOQ), relative standard deviation (RSD), and recovery for all validated toxins in blank matrix

Analyte	LOD	LOQ	Precision (RSD) [%]			Recovery [%]			
	[µg/kg]	[µg/kg]	Inter-injection (*n* = 10)	Intra-day (*n* = 3)	Inter-day (*n* = 9)	Level 1	Level 2	Level 3	Level 4
DON	1.22	3.61	1.26	3.26	2.82	112	115	118	120
D3G	14.1	41.9	9.92	9.08	4.44	101	87.0	77.8	79.7
3-AcDON	0.96	2.84	1.26	3.50	12.5	94.2	84.7	81.6	73.9
15-AcDON	1.05	3.10	2.87	3.68	6.57	101	103	108	93.2
HT-2	0.27	0.78	3.60	1.05	4.12	120	112	85.4	85.2
T-2	0.07	0.21	2.58	4.67	9.73	119	101	91.7	95.6
AOH	0.94	2.77	2.43	1.81	8.91	112	112	113	108
AME	0.05	0.17	5.06	5.44	4.48	113	100	105	115
TeA	0.30	0.90	2.37	2.40	7.28	78.4	100	98.1	98.9
AFB1	0.02	0.05	3.00	8.26	7.07	116	86.4	89.4	114
AFB2	0.01	0.04	2.05	7.00	7.73	96.3	95.3	90.8	98.6
AFG1	0.02	0.07	2.04	3.04	8.28	96.3	99.8	100	103
AFG2	0.28	0.84	3.68	8.99	5.69	97.4	92.1	96.7	98.7
STC	0.01	0.02	3.47	2.14	9.20	119	105	98.9	75.9
OTA	0.94	2.79	2.19	5.27	9.27	81.1	72.9	70.3	119

#### LODs and LOQs

The LODs and LOQs were determined according to the protocol of Vogelgesang and Hädrich (1998). Therefore, the analyte-free blank matrix was spiked in triplicate at four different concentration levels. The LODs ranged between 0.01 µg/kg (AFB2 and STC) and 14.1 µg/kg (D3G), while the LOQs ranged between 0.02 µg/kg (STC) and 41.9 µg/kg (D3G). Thus, D3G showed the highest LOD and LOQ of all mycotoxins. Without D3G, the LODs were between 0.01 µg/kg (AFB2 and STC) and 1.22 µg/kg (DON), while the LOQs were between 0.02 µg/kg (STC) and 3.61 µg/kg (DON).

A possible explanation for the relatively high LOD and LOQ of D3G could be low ionization efficiency, and that it may partially dissolve in the water phase during the QuEChERS cleanup process. In previous studies, this toxin already exhibited the highest LOD and LOQ compared to the other analytes (Scheibenzuber et al. 2022; Biehl et al. 2024; Dick et al. 2024).

Apart from that, a comparison of the determined LODs and LOQs of the other analytes with the values from recent studies by our group shows comparable or slightly higher limits (Scheibenzuber et al. 2022; Biehl et al. 2024; Dick et al. 2024). However, all these previous LODs and LOQs were determined for cereal products with a starch matrix. Therefore, the slightly higher LODs and LOQs in this study could be attributed to the more complex matrix used. When comparing the obtained LODs and LOQs with those from the literature for fat-rich matrices, the limits determined in this study are significantly lower (Njumbe Ediage et al. 2011; Abia et al. 2013; Cunha et al. 2018; Mihalache et al. 2024).

#### Recovery

The analyte-free blank matrix was spiked in triplicate at four different concentration levels to determine the recovery. The selected concentrations reflect typical levels expected in naturally contaminated samples. A relatively high concentration was chosen as the highest level to receive a wide working range. The recovery rates all range between 70.3% (OTA) and 120% (HT-2), falling within the required range of 70–120% according to Vogelgesang and Hädrich (1998).

#### Precision

To receive the inter-injection, the inter-day, and the intra-day precision, each analyte’s relative standard deviation was calculated after a specific number of measurements. A mixture of all analytes diluted in MeOH/H_2_O (60/40, v/v) was injected ten times in a row into the LC-MS/MS system to determine the inter-injection precision. Values of 1.26% (DON) – 9.92% (D3G) were achieved, confirming the LC-MS/MS system’s stability. To determine the intra-day precision (*n* = 3), an analyte-free blank matrix was spiked at one concentration level in triplicate. For inter-day precision (*n* = 9), the blank matrix was spiked in triplicate at one concentration on three different days within three weeks. The obtained precisions were all below 13%, confirming the method’s good precision.

#### Trueness

The accuracy of the method was confirmed for AFB1, AFB2, and AFG1 by analyzing a certified reference material (defatted peanut meal) containing 17.1 ± 2.4 µg/kg AFB1, 3.0 ± 0.4 µg/kg AFB2, and 3.0 ± 0.5 µg/kg AFG1. In our analysis, we obtained values of 19.1 ± 0.36 µg/kg AFB1 (bias: +11.7%), 3.36 ± 0.08 µg/kg AFB2 (bias: +12.0%), and 3.11 ± 0.21 µg/kg AFG1 (bias: +3.7%), which are therefore all within the specified certified range.

Considering all validation results, the method can be regarded as satisfactory. Thus, it is suitable for simultaneously analyzing 15 mycotoxins in fatty matrices.

### Analysis of commercial PBAPs

**Fig. 2:**
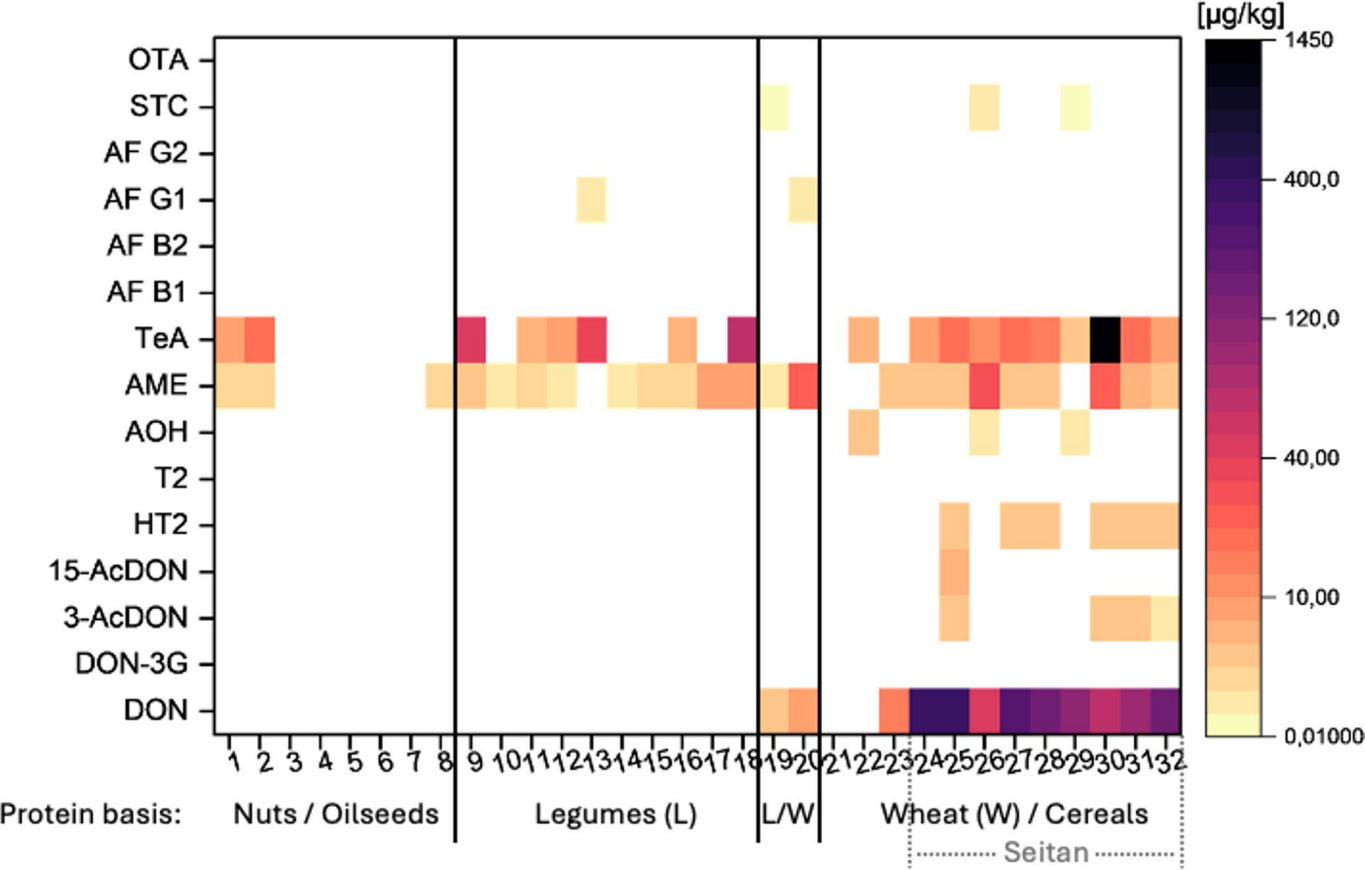
Occurrence and mean concentration (> LOQ) of mycotoxins in plant-based meat, cheese, and fish alternatives

In total, thirty-two PBMAs (*n* = 26), PBCAs (*n* = 4), and PBFAs (*n* = 2) were purchased from German and Belgian supermarkets and analyzed for their mycotoxin content to determine the contamination of supermarket products with *Fusarium*, *Alternaria*, and *Aspergillus* toxins. An overview of all products analyzed in this study can be found in Supplementary Table 1 in the SI. The results are presented in Fig. 2. The analyzed products can be categorized into different groups based on their protein source. The categories include nuts and oilseeds, legumes, legumes mixed with wheat, as well as wheat and other cereals. Since mycotoxin contamination in soy-based PBMAs has already been extensively studied in the literature (Schollenberger et al. 2007; Rodríguez-Carrasco et al. 2019; Oleynikova et al. 2020; Tian et al. 2022), we largely omitted the analysis of soy products. Only one legume-based product (sample no. 18 – a vegan replacement for gyros) was soy-based, and product no. 20 (a vegetarian replacement for a chicken fillet) was based on a mix of wheat and soy protein. As an alternative to soy, pea protein has been gaining increasing importance. Therefore, nearly all other analyzed products in the legume protein category are based on pea protein. Additionally, we focused on seitan products, as seitan plays a crucial role in the world of PBMAs. As previously mentioned, mycotoxin contamination in seitan has been scarcely studied so far. Therefore, we included nine seitan samples (samples no. 24–32) in our study.

As anticipated, certain mycotoxins were closely linked to the plant-based origin of the alternative. This will be further explained when discussing the individual PBMA categories. The average concentrations presented in the following (Table 3 and Supplementary Tables 2-6 in the SI) represent the mean of all samples exceeding the LOQ. In contrast, the exposure assessment was conducted using the LB and UB approach, as described in the methods section. All concentrations refer to the fresh weight.

**Table 3:** Mycotoxin content in 32 plant-based alternative products (PBAPs)

Analyte	Number of samples > LOQ	Percentage [%] of samples > LOQ	Mean concentration of all samples > LOQ [µg/kg]	Maximum concentration [µg/kg]
DON	12	37.5	125	333
D3G	0	0	0	0
3-AcDON	4	12.5	2.41	3.82
15-AcDON	1	3.13	6.77	6.77
HT-2	6	18.8	2.96	4.40
T-2	0	0	0	0
AOH	3	9.38	1.47	2.80
AME	23	71.9	5.61	28.0
TeA	18	56.3	96.2	1430
AFB1	0	0	0	0
AFB2	0	0	0	0
AFG1	2	6.25	0.39	0.65
AFG2	0	0	0	0
STC	3	9.38	0.08	0.13
OTA	0	0	0	0

#### General remarks

In general, it could be observed that while D3G, AFB1, AFB2, AFG2, and OTA were not detected in any of the products, the *Alternaria* toxins AME and TeA were the most frequently found mycotoxins. AME was present in 72% of the samples, with a maximum concentration of 28 µg/kg (in a seitan product), while TeA was detected in 56% of the samples, reaching a maximum concentration of 1,430 µg/kg (in another seitan product).

The most frequently detected *Fusarium* toxin was DON, which was found in 38% of the samples, with a maximum concentration of 333 µg/kg in a wheat-based sausage. The *Aspergillus* toxins STC and AFG1 were detected in 6% and 9% of the samples, with maximum levels of 0.65 µg/kg (AFG1 in a legume- and wheat-based alternative for chicken fillet) and 0.13 µg/kg (STC in a natural seitan product), respectively.

#### *Fusarium* toxins

The *Fusarium* toxin DON was not detectable in any of the products based on nuts and oilseeds or legume-based products. However, it was present in almost all wheat- and cereal-based products, regardless of whether they were combined with legumes or not. The average concentration of DON in wheat-based products was 148 µg/kg, and in the wheat and legume-based products, it was 6.60 µg/kg. A closer look at the wheat-based products revealed that the highest DON concentrations were found in seitan products. In this category, DON was detectable in all nine products, with an average concentration of 164 µg/kg and a maximum concentration of 333 µg/kg.

Only one product (a salmon alternative) did not contain detectable levels of DON (< LOD). This particular product was the only one that did not contain wheat protein but was instead based on starch and rice. In one wheat-based product, DON was detected but could not be quantified (< LOQ).

All other detected *Fusarium* toxins were also exclusively found in wheat-containing products. The DON derivatives 3-AcDON and 15-AcDON were detected solely in seitan products. The average concentration of 3-AcDON in the four seitan products containing this toxin was 2.41 µg/kg, while 15-AcDON was found in one seitan product at a level of 6.77 µg/kg.

The trichothecene HT-2 was detected in six products with an average concentration of 2.96 µg/kg. All six of these positive findings were in seitan products.

As mentioned before, no data could be found regarding the contamination of seitan with mycotoxins. Mihalache et al. (2023) concluded in their study that all analyzed samples, except for seitan products, contained at least one mycotoxin. However, their study included only one seitan product and focused solely on zearalenone and fumonisins as the only determined *Fusarium* toxins. DON and other trichothecenes were omitted. In a more recent study, Mihalache et al. (2024) could determine DON in wheat PBMAs but did not specify if their study included seitan products or if other wheat PBMAs were analyzed. They found concentrations ranging between 25.5 and 89.1 µg/kg (DON) and 12.8–93.6 µg/kg (HT-2) in their wheat PBMAs and even higher trichothecene concentrations (22.4–106 µg/kg DON, 8.5–158 µg/kg HT-2, and 3.3–18.5 µg/kg T-2) in legume PBMAs. Our results highlight the necessity of including seitan products in analyzing PBAPs, with a particular focus on *Fusarium* toxins and trichothecenes.

#### *Aspergillus* toxins

The *Aspergillus* toxins AFG1 and STC were only present in five of the analyzed products. As mentioned earlier, AFB1, AFB2, AFG2, and OTA were not detected in any of the products. STC was found in two seitan products and one product based on a wheat-legume (pea) mix, with a mean concentration of 0.08 µg/kg. AFG1 was detected in two legume-containing samples: one was a pea protein product, and the other was based on a mix of wheat and soy. The mean concentration was 0.39 µg/kg.

Mihalache et al. (2024) also did not detect AFB2 in over 100 analyzed PBMAs. However, unlike our findings, they found AFB1 in legume-based PBMAs with concentrations ranging from 1.60 to 1.92 µg/kg. It is not further specified in which legume-based products the AFB1 was detected or whether these products were soy-based PBMAs. Existing literature has already reported AFB1 in soy burgers, with concentrations of 2.40 µg/kg (Mihalache et al. 2023) and 10.1 µg/kg (Rodríguez-Carrasco et al. 2019). Additionally, Mihalache et al. (2024) detected AFG1 and AFG2 in the analyzed products across all their product categories, with values ranging from 3.2 to 63.7 µg/kg, while Mihalache et al. (2023) found AFG1 in a wheat- and chickpea-based sliced meat. Rodríguez-Carrasco et al. (2019) did not detect AFB2, AFG1, or AFG2 in their analyzed soy-based burgers.

#### *Alternaria* toxins

The *Alternaria* toxins AME and TeA were the most frequently detected mycotoxins. Notably, their occurrence appears to be independent of the protein source. Overall, their average concentrations were 5.61 µg/kg (AME) and 96.2 µg/kg (TeA).

AME was quantified in samples based on nuts and oilseeds as follows: in three out of eight samples of this product category, an average concentration of 1.63 µg/kg was found. All three products with a positive detection were based on sunflower seeds. Four of the five samples that did not contain AME were PBCAs made from walnut, almond, or cashew proteins. In the same product category, TeA was found in only two investigated samples, with an average concentration of 13.1 µg/kg. Both products were based on sunflower seeds.

Looking at products based on legumes, it was visible that nearly every product contained AME. The average concentration of this toxin in legume-based samples was 2.72 µg/kg. Only one product (based on pea protein) did not contain AME. TeA was present in six legume-protein-based samples, with a mean concentration of 28.7 µg/kg. The two investigated samples, which were based on a wheat-legume protein mix, contained AME with a mean concentration of 11.9 µg/kg but did not contain TeA.

Taking a closer look at the products based on wheat and cereals showed that the *Alternaria* toxins had the highest concentrations. AME was detectable in nine of 12 products in this category, with a mean concentration of 8.43 µg/kg. The product with the highest AME concentration of all samples (28.0 µg/kg) was a natural seitan product. Nearly all seitan products (eight out of ten) contained AME. The mean concentration was 9.00 µg/kg when considering only seitan products. The same pattern was observed for TeA. The mean concentration of this toxin across the ten wheat-based products was 153 µg/kg. The product with the highest TeA concentration was a sausage made from seitan (1,430 µg/kg). All nine observed seitan products contained TeA, and the average concentration of TeA in the seitan products was 170 µg/kg.

The *Alternaria* toxin AOH was found only in the wheat- and cereal-based product category, with an average concentration of 1.47 µg/kg. AOH was detected in three wheat-based products: two were seitan products, and one was a wheat-based substitute for cured sausage, which contained the highest concentration of the three products (2.80 µg/kg).

These results also highlight the necessity of including seitan products while investigating the *Alternaria* mycotoxin contamination of PBAPs.

Mihalache et al. (2024) also detected *Alternaria* toxins in all analyzed PBAPs (made from wheat, legumes, and vegetables) with values ranging between 3.10 and 8.80 µg/kg (AME) and 3.90–153.8 µg/kg for AOH. So, they determined higher concentrations of AOH than we did, but lower values of AME in wheat- and legume-based PBMAs compared to our findings. Similar values were observed in the study by Mihalache et al. (2023), which reported concentrations of 0.30–12.1 µg/kg for the analyzed *Alternaria* toxins. Higher concentrations were found by Rodríguez-Carrasco et al. (2019) in their analysis of soy-based burgers, with values ranging between 179 and 408 µg/kg (AME) and 185 µg/kg (AOH, only found in one sample).

#### Summary of contamination situation

In principle, at least one out of 15 mycotoxins was detected in 27 of 32 plant-based alternative products, with a maximum of six combined mycotoxins. The only products that showed no contamination were the four cheese alternatives based on nut protein and one fish alternative made from starch and rice. Seitan products were identified as particularly affected by mycotoxin contamination.

### Risk assessment of PBAPs

To assess the risk for consumers due to mycotoxins in PBAPs, the LB, MB, and UB values were first calculated, followed by the estimation of the EDI. To evaluate the risk for different population groups, we distinguished between toddlers, preschoolers, adolescents, adults, and elderly people. The results are summarized in Tables 4, 5 and 6. In the risk assessment, we decided not to differentiate between the previously defined product categories (wheat-based alternatives, legume-based alternatives, etc.), as consumers tend to mix different product groups when replacing animal-based products. However, due to the outstanding role of seitan in the quantitative data, we chose to conduct a separate risk assessment for seitan products in addition to the general evaluation. All calculations were based on the respective HBGV (TDI, TTC) as mentioned before, and, when available, on government-established maximum limits for relevant food groups.

**Table 4:** Estimated daily intake (EDI) [µg/kg bw x day] and hazard quotient (HQ) [%] calculated using the lower bound scenario (LB)

	Toddlers (1–3 yrs)				Preschoolers (4–5 yrs)				Adolescents (14–18 yrs)				Adults (19–64 yrs)				Elderly (65–80 yrs)			
	Seitan products		All PBAPs		Seitan products		All PBAPs		Seitan products		All PBAPs		Seitan products		All PBAPs		Seitan products		All PBAPs	
	EDI	HQ	EDI	HQ	EDI	HQ	EDI	HQ	EDI	HQ	EDI	HQ	EDI	HQ	EDI	HQ	EDI	HQ	EDI	HQ
DON	0.47	47.0	0.13	13.5	0.38	38.4	0.11	11.0	0.32	31.9	0.09	9.15	0.27	27.6	0.08	7.90	0.20	20.0	0.06	5.74
D3G	0		0		0		0		0		0		0		0		0		0	
3-AcDON	0.003		9 × 10^− 4^		0.002		7 × 10^− 4^		0.002		6 × 10^− 4^		0.002		5 × 10^− 4^		0.001		4 × 10^− 4^	
15-AcDON	0.002		6 × 10^− 4^		0.002		5 × 10^− 4^		0.001		4 × 10^− 4^		0.001		4 × 10^− 4^		9 × 10^− 4^		3 × 10^− 4^	
HT-2	0.006	28.0	0.002	7.98	0.005	22.9	0.001	6.52	0.004	19.0	0.001	5.42	0.003	16.4	9 × 10^− 4^	4.68	0.002	11.9	7 × 10^− 4^	3.40
T-2	0		0		0		0		0		0		0		0		0		0	
AOH	5 × 10^− 4^	20.1	4 × 10^− 4^	15.6	4 × 10^− 4^	16.5	3 × 10^− 4^	12.7	3 × 10^− 4^	13.7	3 × 10^− 4^	10.6	3 × 10^− 4^	11.8	2 × 10^− 4^	9.14	2 × 10^− 4^	8.58	2 × 10^− 4^	6.64
AME	0.02	907	0.01	457	0.02	742	0.009	374	0.02	616	0.008	311	0.13	532	0.007	268	0.01	387	0.005	195
TeA	0.48	32.1	0.15	10.2	0.39	26.2	0.125	8.36	0.33	21.8	0.10	6.95	0.28	18.8	0.09	6.00	0.20	13.7	0.07	4.36
AFB1	0	-	0	-	0	-	0	-	0	-	0	-	0	-	0	-	0	-	0	-
AFB2	0	-	0	-	0	-	0	-	0	-	0	-	0	-	0	-	0	-	0	-
AFG1	0	-	7 × 10^− 5^	-	0	-	6 × 10^− 5^	-	0	-	5 × 10^− 5^	-	0	-	4 × 10^− 5^	-	0	-	3 × 10^− 5^	-
AFG2	0	-	0	-	0	-	0	-	0	-	0	-	0	-	0	-	0	-	0	-
STC	6 × 10^− 5^	-	2 × 10^− 5^	-	5 × 10^− 5^	-	2 × 10^− 5^	-	4 × 10^− 5^	-	1 × 10^− 5^	-	4 × 10^− 5^	-	1 × 10^− 5^	-	3 × 10^− 5^	-	1 × 10^− 5^	-
OTA	0	-	0	-	0	-	0	-	0	-	0	-	0	-	0	-	0	-	0	-

**Table 5:** Estimated daily intake (EDI) [µg/kg bw x day] and hazard quotient (HQ) [%] calculated using the middle bound scenario (MB)

	Toddlers (1–3 yrs)				Preschoolers (4–5 yrs)				Adolescents (14–18 yrs)				Adults (19–64 yrs)				Elderly (65–80 yrs)			
	Seitan products		All PBAPs		Seitan products		All PBAPs		Seitan products		All PBAPs		Seitan products		All PBAPs		Seitan products		All PBAPs	
	EDI	HQ	EDI	HQ	EDI	HQ	EDI	HQ	EDI	HQ	EDI	HQ	EDI	HQ	EDI	HQ	EDI	HQ	EDI	HQ
DON	0.47	49.2	0.13	15.8	0.38	40.2	0.11	12.9	0.31	33.4	0.09	10.8	0.27	28.9	0.08	9.29	0.20	21.0	0.06	6.75
D3G	0.02		0.02		0.02		0.02		0.01		0.01		0.01		0.01		0.009		0.009	
3-AcDON	0.004		0.002		0.003		0.002		0.003		0.001		0.002		0.001		0.002		0.0009	
15-AcDON	0.003		0.002		0.003		0.002		0.002		0.001		0.002		0.001		0.001		0.0009	
HT-2	0.006	29.1	0.002	10.0	0.005	23.8	0.002	8.19	0.004	19.8	0.001	6.81	0.003	17.1	0.001	5.88	0.002	12.4	0.0008	4.27
T-2	0.0001		0.0001		0.00008		0.00008		0.00007		0.00007		0.00006		0.00006		0.00004		0.00004	
AOH	0.002	61.6	0.002	63.9	0.001	50.3	0.001	52.2	0.001	41.8	0.001	43.4	0.001	36.1	0.0009	37.5	0.0007	26.2	0.0007	27.2
AME	0.02	908	0.01	458	0.02	742	0.009	375	0.02	617	0.008	311	0.01	533	0.007	269	0.01	387	0.005	195
TeA	0.48	32.1	0.15	10.2	0.39	26.2	0.13	8.37	0.32	21.8	0.10	6.96	0.29	18.8	0.09	6.01	0.20	13.7	0.07	4.36
AFB1	0.00003	-	0.00003	-	0.00002	-	0.00002	-	0.00002	-	0.00002	-	0.00002	-	0.00002	-	0.000001	-	0.00001	-
AFB2	0.00001	-	0.00001	-	0.00001	-	0.00001	-	0.00001	-	0.00001	-	0.00001	-	0.000008	-	0.00006	-	0.00006	-
AFG1	0.00003	-	0.0001	-	0.00002	-	0.00008	-	0.00002	-	0.00007	-	0.00002	-	0.00006	-	0.00001	-	0.00004	-
AFG2	0.0004	-	0.0004	-	0.0003	-	0.0003	-	0.0003	-	0.0003	-	0.0002	-	0.0002	-	0.0002	-	0.0002	-
STC	0.00007	-	0.0003	-	0.00006	-	0.00003	-	0.0001	-	0.00002	-	0.00004	-	0.00003	-	0.00003	-	0.00001	-
OTA	0.001	-	0.001	-	0.001	-	0.001	-	0.001	-	0.0009	-	0.0008	-	0.0008	-	0.0006	-	0.0006	-

**Table 6:** Estimated daily intake (EDI) [µg/kg bw x day] and hazard quotient (HQ) [%] calculated using the upper bound scenario (UB)

	Toddlers (1–3 yrs)				Preschoolers (4–5 yrs)				Adolescents (14–18 yrs)				Adults (19–64 yrs)				Elderly (65–80 yrs)			
	Seitan products		All PBAPs		Seitan products		All PBAPs		Seitan products		All PBAPs		Seitan products		All PBAPs		Seitan products		All PBAPs	
	EDI	HQ	EDI	HQ	EDI	HQ	EDI	HQ	EDI	HQ	EDI	HQ	EDI	HQ	EDI	HQ	EDI	HQ	EDI	HQ
DON	0.47	51.4	0.14	18.2	0.38	42.0	0.11	14.9	0.32	34.9	0.09	12.4	0.27	30.2	0.08	10.7	0.20	21.9	0.06	7.77
D3G	0.04		0.04		0.03		0.33		0.03		0.03		0.02		0.02		0.02		0.02	
3-AcDON	0.005		0.003		0.004		0.003		0.003		0.002		0.003		0.002		0.002		0.001	
15-AcDON	0.005		0.003		0.004		0.003		0.003		0.002		0.003		0.002		0.002		0.001	
HT-2	0.006	30.2	0.002	12.2	0.005	24.7	0.002	9.96	0.004	20.5	0.002	8.28	0.003	17.7	0.001	7.15	0.002	12.0	0.001	5.19
T-2	0.0002		0.0002		0.0002		0.0002		0.0001		0.0001		0.0001		0.0001		0.00008		0.00008	
AOH	0.003	103	0.003	112	0.002	84.2	0.002	91.7	0.002	70.0	0.002	76.2	0.002	60.4	0.002	65.8	0.001	43.9	0.001	47.8
AME	0.02	909	0.01	459	0.02	743	0.009	376	0.02	618	0.008	312	0.01	534	0.007	270	0.01	387	0.005	196
TeA	0.48	32.1	0.15	10.3	0.39	26.2	0.13	8.38	0.33	21.8	0.10	6.97	0.28	18.8	0.09	6.02	0.20	13.7	0.07	4.37
AFB1	0.00006	-	0.00006	-	0.00005	-	0.00005	-	0.00004	-	0.00004	-	0.00003	-	0.00003	-	0.00002	-	0.00002	-
AFB2	0.00003	-	0.00003	-	0.00002	-	0.00002	-	0.00002	-	0.00002	-	0.00002	-	0.00002	-	0.00001	-	0.00001	-
AFG1	0.00006	-	0.0001	-	0.00005	-	0.0001	-	0.00004	-	0.00009	-	0.00003	-	0.00007	-	0.00002	-	0.00005	-
AFG2	0.0008	-	0.0008	-	0.0006	-	0.0006	-	0.0005	-	0.0005	-	0.0005	-	0.0005	-	0.0003	-	0.0003	-
STC	0.00009	-	0.00005	-	0.00007	-	0.00004	-	0.00006	-	0.00003	-	0.00005	-	0.00003	-	0.00004	-	0.00002	-
OTA	0.003	-	0.003	-	0.002	-	0.002	-	0.002	-	0.002	-	0.002	-	0.002	-	0.001	-	0.001	-

#### *Fusarium* toxins

When looking at the UB scenario for toddlers as the most vulnerable group, the HQ for the sum of the *Fusarium* toxins DON, 3- and 15-AcDON, and D3G reached 18.2% for all PBAPs, indicating no significant consumer risk. However, the HQ was 51.4% for seitan products for toddlers, which should not be overlooked. For a worst-case scenario, the product with the highest DON concentration (product number 24, DON = 333 µg/kg) was examined in more detail. After considering the UB for AcDONs and D3G, the HQ reached 98.9%, which has to be considered critical. Looking at the Commission Regulation 2024/1022 regarding maximum levels of DON and its derivatives and modified forms in food (European Commission 2024a), PBAPs are not explicitly listed in this regulation. However, the category *“cereal-based foods for infants and young children”* is included with a maximum level of 150 µg/kg DON. None of the analyzed products were labeled as food for young children. However, assuming that wheat-based plant alternatives are also used in the diet of toddlers, a closer look at seitan products reveals that 4 out of 9 of these specific products exceeded 150 µg/kg. Therefore, incorporating seitan products into the diet of young children is not advisable.

When examining the other trichothecenes, T-2 and HT-2, it becomes clear that the HQ does not exceed 12.2% across all analyzed products. For seitan products, the HQ reached 30.2% for toddlers. A comparison with the Commission Regulation 2024/1038 regarding maximum levels of T-2 and HT-2 in food (European Commission 2024b), which sets a maximum value of 10 µg/kg for the sum of HT-2 and T-2 in *“cereal-based foods for infants and young children”*, reveals that none of the seitan products exceed these limits (maximum detected HT-2 = 4.40 µg/kg, with no T-2 detected at all).

#### *Alternaria* toxins

However, the situation becomes dramatic when considering *Alternaria* toxins. Regardless of whether the LB, MB, or UB scenario is applied, replacing animal-based products with PBAPs leads to the EDI of AME exceeding the TTC significantly across all age groups.

In toddlers, the EDI exceeded the TTC of AME by a factor of around 4.6 for all scenarios. This factor decreased with age due to higher bw and was around 3.7 in preschoolers, around 3.1 in adolescents, and around 2.7 in adults. The lowest exceedance was observed in the elderly due to less consumption, where the EDI surpassed the TTC still by a factor of around 1.9. The concern intensified when focusing specifically on seitan products. In toddlers, the EDI exceeded the TTC by a factor of around 9.1, independent of the bound scenario used. For preschoolers, this factor was around 7.4, for adolescents around 6.2, for adults around 5.3, and for elderly individuals around 3.9. The potential risk became even more evident when considering the product with the highest AME concentration. In this worst-case scenario, the EDI exceeded the TTC by a factor of 31.7 in toddlers, 25.9 in preschoolers, 21.5 in adolescents, 18.6 in adults, and 13.5 in the elderly. Despite the toxicological weakness of the TTC concept, this finding was very alarming.

However, the concern regarding *Alternaria* toxins was mainly relevant to AME. The HQ for TeA remained at a maximum of 10.3% across all examined products and 32.1% for seitan products, both values accounting for the UB scenario for toddlers. For AOH, the maximum HQ (UB scenario for toddlers) reached 112**%** (all products) and 103**%** (seitan products). Since AOH was detected in only a limited number of products (in 3/32 products in general and 3/12 seitan samples), there is a substantial difference between the LB, MB, and UB exposure scenarios. In the LB scenario, the maximum utilization (toddlers) of the TTC is 20.1% for seitan products and 15.6% for all products, and in the MB scenario, it was still 20.1% for seitan products, but 61.6% for all products. The products with the highest detected concentrations of TeA (sample no. 30: 1,430 µg/kg TeA) or AOH (sample no. 22: 2.8 µg/kg AOH) would result in a maximum TTC utilization of 270% (TeA) or 317% (AOH) for toddlers. These values were still critical from an individual point of view, but the risk was not as universally pronounced as for AME.

Although there are no government-regulated maximum limits for *Alternaria* toxins, the European Union issued recommendations for monitoring the presence of *Alternaria* toxins in food (European Commission 2022). While PBAPs are not explicitly listed in this recommendation, the category *“cereal-based foods for infants and young children”* is included. The recommendation states that products should not contain more than 2 µg/kg of AME or AOH and no more than 500 µg/kg of TeA.

Since all products with the highest detected values are wheat-based (sample no. 22, with maximum AOH content) or seitan products (sample no. 26, with maximum AME content, and sample no. 30, with maximum TeA content), this recommendation can be used to assess these products. The concentrations of *Alternaria* toxins in the three products mentioned before exceeded the recommended maximum levels, meaning these products should not be used as food for toddlers. While no other product exceeds the recommended value for AOH and TeA, twelve additional PBAPs contained concentrations above 2 µg/kg AME. When focusing on seitan products, it became evident that 8 out of the 9 analyzed samples (including 7 additional products alongside sample no. 26) exceeded 2 µg/kg. This emphasizes again that caution should be exercised when using seitan products in the diet of young children.

#### *Aspergillus* toxins

Regarding *Aspergillus* toxins, AFB1 is the only aflatoxin for which a BMDL_10_ exists. Since we could only find AFG1 and STC in our samples, but no AFB1, we could not determine a realistic MoE. When the LODs of the method for AFB1 were used for calculation, MoEs greater than 10,000 were observed for elderly individuals (16,568), adults (12,030), and adolescents (10,389). However, MoEs below 10,000 were found for toddlers (7,058) and preschoolers (8,635) when the LOD of the method is used. This concept is therefore not applicable to our study, as our method was not sensitive enough.

Assessing the detected AFG1 concentrations using the Commission Regulation 2023/915 regarding maximum levels for certain contaminants in food (European Commission 2023) did not prove successful, as this regulation does not explicitly list PBAPs as a food category. All products in which AFG1 was detected are based on legume proteins, a category that is also not covered by the regulation. The same problem is encountered for seitan products. The regulation only specifies a maximum level of 0.1 µg/kg AFB1 for cereal-based infant food but does not limit the total sum of aflatoxins. Since AFB1 was not detected in any product, an assessment based on this regulation is not possible.

## Conclusion

A quantitative analysis of the mycotoxin content in PBAPs revealed that contamination was both universal and pronounced. While *Alternaria* toxins were present across all product groups, *Aspergillus* toxins were detected only sporadically, and *Fusarium* toxins were found exclusively in wheat-based alternatives. 29 out of 32 products showed contamination with at least one mycotoxin. Seitan products were particularly affected by contamination with both *Fusarium* and *Alternaria* toxins. These findings highlight the relevance of mycotoxin contamination in the investigated products. However, this is only a part of the real situation. To assess the safety of plant-based alternatives in general, additional aspects such as pesticide residues, heavy metal contamination, processing contaminants, and the nutritional composition of the products would need to be evaluated. Due to the limited number of products included in this study and restricted analytical capacities, such further investigations are beyond the scope of the present work and will require support from official food control authorities.

To assess the risk for different population groups, we conducted a risk assessment for toddlers, preschoolers, adolescents, adults, and elderly people for the consumption of PBAPs in general and for seitan products specifically.

Our study revealed a potential risk, especially for toddlers, but also for individuals of all ages, due to *Alternaria* toxins. This risk exists regardless of the product category, but is particularly concerning in the case of seitan products.

Therefore, we conclude that especially seitan products must be systematically monitored for their mycotoxin content in the future, with special attention to *Alternaria* toxins, even though no governmental maximum levels have been established for them yet. Moreover, additional toxicological studies on *Alternaria* toxins are urgently needed, as TTC-based risk assessment is not ideal, and TDIs would enable better evaluation of their relevance in these products. However, besides authorities and toxicologists, manufacturers can also contribute to the safety of these products, and future research should explore key ingredients contributing to contamination with *Alternaria* toxins and processing steps to minimize their transfer. In the meantime, these products should not be included in the diet of toddlers.

## Supplementary information

Below is the link to the electronic supplementary material.

Supplementary Material 1 (DOCX 58.3 KB)

## Data Availability

Data are provided within the manuscript and the Supplementary Information files. Raw data are available from the corresponding author upon reasonable request.

## Abbreviations

15-AcDON: 15-Acetyldeoxynivalenol
3-AcDON: 3-Acetyldeoxynivalenol
AF: Aflatoxin
AME: Alternariolmonomethylether
AOH: Alternariol
bw: Body weight
DON: Deoxynivalenol
D3G: Deoxynivalenol-3-glucoside
EDI: Estimated daily intake
HQ: Hazard quotient
HBGV: Health-based guidance values
HT-2: HT-2 toxin
LC-MS/MS: Liquid chromatography-tandem mass spectrometry
LOD: Limit of detection
LOQ: Limit of quantitation
LB: Lower bound
MB: Middle bound
MoE: Margin of exposure
OTA: Ochratoxin A
PBAP: Plant-based alternative product
PBCA: Plant-based cheese alternative
PBFA: Plant-based fish alternative
PBMA: Plant-based meat alternative
SIDA: Stable isotope dilution assay
STC: Sterigmatocystin
T-2: T-2 toxin
TeA: Tenuazonic acid
TDI: Tolerable daily intake
TTC: Threshold for toxicological concern
UB: Upper bound
