# Supplementary material for: Mycotoxin occurrence and risk assessment in plant-based meat, cheese, and fish alternatives based on an adapted UHPLC-MS/MS multi-method
Source: Mycotoxin Res. 2026 Feb 3;42(1):22. doi: 10.1007/s12550-026-00636-2 (PMC12868108; doi:10.1007/s12550-026-00636-2)
Supplement: Supplementary file 1 — Supplementary Material 1 (DOCX 58.3 KB) [file 12550_2026_636_MOESM1_ESM.docx]

**Supplementary Information**

Supplementary Table 1: Overview of the plant-based meat, cheese, and fish alternatives from this study

Supplementary Table 2: Occurrence and concentration of mycotoxins in the plant-based meat, cheese, and fish alternatives

Supplementary Table 3: Mycotoxin content in 8 alternative products based on nuts and oilseeds

Supplementary Table 4: Mycotoxin content in 10 alternative products based on legumes

Supplementary Table 5: Mycotoxin content in 2 alternative products based on legumes mixed with wheat

Supplementary Table 6: Mycotoxin content in 8 alternative products based on wheat and cereals

Supplementary Table 7: Lower bound (< NWG = 0; < BG = 0), middle bound (< NWG = ½ NWG; < BG = ½ BG), and upper bound (< NWG = NWG, < BG = BG) scenarios for each mycotoxin, calculated for all plant-based alternative products and all seitan products

Supplementary Table 8: Detailed spiking information for the determination of the recovery

Supplementary Table 9: Detailed spiking information for the determination of LODs and LOQs

Supplementary Table 1: Overview of the plant-based meat, cheese, and fish alternatives from this study

| Product number | Product description | Protein basis |
| --- | --- | --- |
| 1 | Vegan replacement for a scalded pork sausage (Mortadella style) with vegetables | Sunflower Seeds |
| 2 | Vegan replacement for a scalded pork sausage (Mortadella style) with pepper | Sunflower Seeds |
| 3 | Vegan replacement for a mild cheese | Cashews |
| 4 | Vegan replacement for a savoury cheese | Walnuts |
| 5 | Vegan replacement for a mild cheese | Almonds |
| 6 | Vegan replacement for a savoury cheese | Almonds |
| 7 | Vegan replacement for meat salad | Sunflower flour & rapeseed oil |
| 8 | Vegan replacement for minced meat | Sunflower seeds |
| 9 | Vegan replacement for hamburger patties/meatballs | Quinoa |
| 10 | Vegan replacement for hamburger patties | Peas |
| 11 | Vegan replacement for a spreadable German cured sausage | Peas |
| 12 | Vegan replacement for cevapcici | Peas |
| 13 | Vegan replacement for minced meat | Peas |
| 14 | Vegan replacement for a fine liver sausage | Peas |
| 15 | Vegan replacement for a traditional Bavarian white sausage ("Weisswurst") | Peas |
| 16 | Vegan replacement for a scalded pork sausage (Bologna style) | Fava beans & peas |
| 17 | Vegan replacement for roast beef | Peas |
| 18 | Vegan replacement for gyros | Soy |
| 19 | Vegan replacement for tuna | Peas & wheat |
| 20 | Vegetarian replacement for a chicken fillet | Soy & wheat |
| 21 | Vegan replacement for smoked salmon | Starch & rice |
| 22 | Vegan replacement for bacon strips | Wheat |
| 23 | Vegetarian replacement for a cured sausage (salami style) | Wheat |
| 24 | Vegan replacement for German-style sausages ("Bratwurst") | Wheat (seitan) |
| 25 | Vegan replacement for roast | Wheat (seitan) |
| 26 | Vegan replacement for meat | Wheat (seitan) |
| 27 | Vegan replacement for meat | Wheat (seitan) |
| 28 | Vegan replacement for minced meat | Wheat (seitan) |
| 29 | Vegan replacement for marinated meat | Wheat (seitan) |
| 30 | Vegan replacement for a cured sausage (salami style) | Wheat (seitan) |
| 31 | Vegan replacement for bacon | Wheat (seitan) |
| 32 | Vegan replacement cold cuts | Wheat (seitan) |

Supplementary Table 2: Occurrence and concentration of mycotoxins in the plant-based meat, cheese, and fish alternatives

|  | Mean concentration ± STD [µg/kg] | | | | | | | | | | | | | | |
| --- | --- | --- | --- | --- | --- | --- | --- | --- | --- | --- | --- | --- | --- | --- | --- |
| Product number | DON | D3G | 3-AcDON | 15-AcDON | HT-2 | T-2 | AOH | AME | TeA | AFB1 | AFB2 | AFG1 | AFG2 | STC | OTA |
| 1 | <LOD | <LOD | <LOD | <LOD | <LOD | <LOD | <LOD | 1.89±0.09 | 8.77±0.39 | <LOD | <LOD | <LOD | <LOD | <LOD | <LOD |
| 2 | <LOD | <LOD | <LOD | <LOD | <LOD | <LOD | <LOD | 1.79±0.07 | 17.5±0.44 | <LOD | <LOD | <LOD | <LOD | <LOD | <LOD |
| 3 | <LOD | <LOD | <LOD | <LOD | <LOD | <LOD | <LOD | <LOD | <LOD | <LOD | <LOD | <LOD | <LOD | <LOD | <LOD |
| 4 | <LOD | <LOD | <LOD | <LOD | <LOD | <LOD | <LOD | <LOD | <LOD | <LOD | <LOD | <LOD | <LOD | <LOD | <LOD |
| 5 | <LOD | <LOD | <LOD | <LOD | <LOD | <LOD | <LOD | <LOD | <LOD | <LOD | <LOD | <LOD | <LOD | <LOD | <LOD |
| 6 | <LOD | <LOD | <LOD | <LOD | <LOD | <LOD | <LOD | <LOD | <LOD | <LOD | <LOD | <LOD | <LOD | <LOD | <LOD |
| 7 | <LOD | <LOD | <LOD | <LOD | <LOD | <LOD | <LOD | <LOQ | <LOD | <LOD | <LOD | <LOD | <LOD | <LOD | <LOD |
| 8 | <LOD | <LOD | <LOD | <LOD | <LOD | <LOD | <LOD | 1.20±0.13 | <LOD | <LOD | <LOD | <LOD | <LOD | <LOD | <LOD |
| 9 | <LOD | <LOD | <LOD | <LOD | <LOD | <LOD | <LOD | 2.24±0.11 | 48.9±1.76 | <LOD | <LOD | <LOD | <LOD | <LOD | <LOD |
| 10 | <LOQ | <LOD | <LOD | <LOD | <LOD | <LOD | <LOD | 0.31±0.01 | <LOD | <LOD | <LOD | <LOD | <LOD | <LOD | <LOD |
| 11 | <LOD | <LOD | <LOD | <LOD | <LOD | <LOD | <LOD | 1.52±0.08 | 6.81±0.21 | <LOD | <LOD | <LOD | <LOD | <LOD | <LOD |
| 12 | <LOD | <LOD | <LOD | <LOD | <LOD | <LOD | <LOD | 0.28±0.01 | 9.95±0.30 | <LOD | <LOD | <LOD | <LOD | <LOD | <LOD |
| 13 | <LOD | <LOD | <LOD | <LOD | <LOD | <LOD | <LOD | <LOD | 39.1±17.7 | <LOD | <LOD | 0.13±0.02 | <LOD | <LOD | <LOD |
| 14 | <LOD | <LOD | <LOD | <LOD | <LOD | <LOD | <LOD | 0.48±0.06 | <LOD | <LOD | <LOD | <LOD | <LOD | <LOD | <LOD |
| 15 | <LOD | <LOD | <LOD | <LOD | <LOD | <LOD | <LOD | 1.30±0.15 | <LOD | <LOD | <LOD | <LOD | <LOD | <LOD | <LOD |
| 16 | <LOD | <LOD | <LOD | <LOD | <LOD | <LOD | <LOD | 1.13±0.10 | 6.94±0.39 | <LOD | <LOD | <LOD | <LOD | <LOD | <LOD |
| 17 | <LOD | <LOD | <LOD | <LOD | <LOD | <LOD | <LOD | 8.34±0.72 | <LOD | <LOD | <LOD | <LOD | <LOD | <LOD | <LOD |
| 18 | <LOD | <LOD | <LOD | <LOD | <LOD | <LOD | <LOD | 8.85±0.48 | 60.5±3.57 | <LOD | <LOD | <LOD | <LOD | <LOD | <LOD |
| 19 | 4.55±1.79 | <LOD | <LOD | <LOD | <LOQ | <LOD | <LOD | 0.34±0.08 | <LOQ | <LOD | <LOD | <LOQ | <LOD | 0.04±0.01 | <LOD |
| 20 | 8.64±0.59 | <LOD | <LOD | <LOD | <LOD | <LOD | <LOD | 23.5±0.28 | <LOD | <LOD | <LOD | 0.65±0.05 | <LOD | <LOD | <LOD |
| 21 | <LOD | <LOD | <LOD | <LOD | <LOD | <LOD | <LOD | <LOD | <LOD | <LOD | <LOD | <LOD | <LOD | <LOD | <LOD |
| 22 | <LOQ | <LOD | <LOD | <LOD | <LOD | <LOD | 2.80±0.46 | <LOD | 6.40±0.27 | <LOD | <LOD | <LOD | <LOD | <LOD | <LOD |
| 23 | 12.3±0.91 | <LOD | <LOD | <LOD | <LOD | <LOD | <LOD | 3.82±0.68 | <LOD | <LOD | <LOD | <LOD | <LOD | <LOD | <LOD |
| 24 | 333±14.2 | <LOD | <LOD | <LOD | <LOD | <LOD | <LOD | 3.98±0.27 | 7.91±0.61 | <LOD | <LOD | <LOD | <LOD | <LOD | <LOD |
| 25 | 332±8.63 | <LOD | 3.82±0.15 | 6.77±0.99 | 3.03±0.50 | <LOD | <LOD | 2.20±0.20 | 18.2±2.78 | <LOD | <LOD | <LOD | <LOD | <LOD | <LOD |
| 26 | 46.0±8.86 | <LOD | <LOD | <LOD | <LOD | <LOD | 0.83±0.10 | 28.0±1.36 | 10.7±0.631 | <LOD | <LOD | <LOD | <LOD | 0.13±0.012 | <LOD |
| 27 | 201±3.12 | <LOD | <LOD | <LOD | 2.09±0.15 | <LOD | <LOD | 2.52±0.24 | 16.2±1.02 | <LOD | <LOD | <LOD | <LOD | <LOD | <LOD |
| 28 | 151±5.99 | <LOD | <LOD | <LOD | 2.18±0.21 | <LOD | <LOD | 2.38±0.15 | 15.0±0.66 | <LOD | <LOD | <LOD | <LOD | <LOD | <LOD |
| 29 | 100±0.62 | <LOD | <LOD | <LOD | <LOD | <LOD | 0.77±0.14 | <LOQ | 2.05±0.19 | <LOD | <LOD | <LOD | <LOD | 0.07±0.00 | <LOD |
| 30 | 66.6±0.44 | <LOD | 2.74±0.39 | <LOD | 4.40±0.30 | <LOD | <LOD | 23.8±1.78 | 1430±26.8 | <LOD | <LOD | <LOD | <LOD | <LOD | <LOD |
| 31 | 90.6±1.67 | <LOD | 2.43±0.74 | <LOD | 3.62±0.52 | <LOD | <LOD | 5.30±0.51 | 17.8±0.68 | <LOD | <LOD | <LOD | <LOD | <LOD | <LOD |
| 32 | 157±4.73 | <LOD | 0.64±0.14 | <LOD | 2.44±0.16 | <LOD | <LOD | 3.86±0.51 | 9.64±0.92 | <LOD | <LOD | <LOD | <LOD | <LOD | <LOD |

Supplementary Table 3: Mycotoxin content in 8 alternative products based on nuts and oilseeds

| Analyte | Number of  samples > LOQ | Percentage [%] of samples > LOQ | Mean concentration of all samples > LOQ [µg/kg] | Maximum concentration [µg/kg] |
| --- | --- | --- | --- | --- |
| DON | 0 | 0.00 | 0.00 | 0.00 |
| D3G | 0 | 0.00 | 0.00 | 0.00 |
| 3-AcDON | 0 | 0.00 | 0.00 | 0.00 |
| 15-AcDON | 0 | 0.00 | 0.00 | 0.00 |
| HT-2 | 0 | 0.00 | 0.00 | 0.00 |
| T-2 | 0 | 0.00 | 0.00 | 0.00 |
| AOH | 0 | 0.00 | 0.00 | 0.00 |
| AME | 3 | 37.5 | 1.63 | 1.89 |
| TeA | 2 | 25.0 | 13.1 | 17.5 |
| AFB1 | 0 | 0.00 | 0.00 | 0.00 |
| AFB2 | 0 | 0.00 | 0.00 | 0.00 |
| AFG1 | 0 | 0.00 | 0.00 | 0.00 |
| AFG2 | 0 | 0.00 | 0.00 | 0.00 |
| STC | 0 | 0.00 | 0.00 | 0.00 |
| OTA | 0 | 0.00 | 0.00 | 0.00 |

Supplementary Table 4: Mycotoxin content in 10 alternative products based on legumes

| Analyte | Number of  samples > LOQ | Percentage [%] of samples > LOQ | Mean concentration of all samples > LOQ [µg/kg] | Maximum concentration [µg/kg] |
| --- | --- | --- | --- | --- |
| DON | 0 | 0.00 | 0.00 | 0.00 |
| D3G | 0 | 0.00 | 0.00 | 0.00 |
| 3-AcDON | 0 | 0.00 | 0.00 | 0.00 |
| 15-AcDON | 0 | 0.00 | 0.00 | 0.00 |
| HT-2 | 0 | 0.00 | 0.00 | 0.00 |
| T-2 | 0 | 0.00 | 0.00 | 0.00 |
| AOH | 0 | 0.00 | 0.00 | 0.00 |
| AME | 9 | 90.0 | 2.72 | 8.85 |
| TeA | 6 | 60.0 | 28.7 | 60.5 |
| AFB1 | 0 | 0.00 | 0.00 | 0.00 |
| AFB2 | 0 | 0.00 | 0.00 | 0.00 |
| AFG1 | 1 | 10.0 | 0.13 | 0.13 |
| AFG2 | 0 | 0.00 | 0.00 | 0.00 |
| STC | 0 | 0.00 | 0.00 | 0.00 |
| OTA | 0 | 0.00 | 0.00 | 0.00 |

Supplementary Table 5: Mycotoxin content in 2 alternative products based on legumes mixed with wheat

| Analyte | Number of  samples > LOQ | Percentage [%] of samples > LOQ | Mean concentration of all  samples > LOQ [µg/kg] | Maximum concentration [µg/kg] |
| --- | --- | --- | --- | --- |
| DON | 2 | 100 | 6.60 | 8.64 |
| D3G | 0 | 0.00 | 0.00 | 0.00 |
| 3-AcDON | 0 | 0.00 | 0.00 | 0.00 |
| 15-AcDON | 0 | 0.00 | 0.00 | 0.00 |
| HT-2 | 0 | 0.00 | 0.00 | 0.00 |
| T-2 | 0 | 0.00 | 0.00 | 0.00 |
| AOH | 0 | 0.00 | 0.00 | 0.00 |
| AME | 2 | 100 | 11.9 | 23.5 |
| TeA | 0 | 0.00 | 0.00 | 0.00 |
| AFB1 | 0 | 0.00 | 0.00 | 0.00 |
| AFB2 | 0 | 0.00 | 0.00 | 0.00 |
| AFG1 | 1 | 50.0 | 0.65 | 0.65 |
| AFG2 | 0 | 0.00 | 0.00 | 0.00 |
| STC | 1 | 50.0 | 0.04 | 0.04 |
| OTA | 0 | 0.00 | 0.00 | 0.00 |

Supplementary Table 6: Mycotoxin content in 12 alternative products based on wheat and cereals

| Analyte | Number of  samples > LOQ | Percentage [%]  of samples > LOQ | Mean concentration of all  samples > LOQ [µg/kg] | Maximum concentration [µg/kg] |
| --- | --- | --- | --- | --- |
| DON | 10 | 83.3 | 148 | 333 |
| D3G | 0 | 0.00 | 0.00 | 0.00 |
| 3-AcDON | 4 | 33.3 | 2.41 | 3.82 |
| 15-AcDON | 1 | 8.33 | 6.77 | 6.77 |
| HT-2 | 6 | 50.0 | 2.96 | 4.40 |
| T-2 | 0 | 0.00 | 0.00 | 0.00 |
| AOH | 3 | 25.0 | 1.47 | 2.80 |
| AME | 9 | 75.0 | 8.43 | 28.0 |
| TeA | 10 | 83.3 | 153 | 1430 |
| AFB1 | 0 | 0.00 | 0.00 | 0.00 |
| AFB2 | 0 | 0.00 | 0.00 | 0.00 |
| AFG1 | 0 | 0.00 | 0.00 | 0.00 |
| AFG2 | 0 | 0.00 | 0.00 | 0.00 |
| STC | 2 | 16.7 | 0.10 | 0.13 |
| OTA | 0 | 0.00 | 0.00 | 0.00 |

Supplementary Table 7: Lower bound (< NWG = 0; < BG = 0), middle bound (< NWG = ½ NWG; < BG = ½ BG), and upper bound (< NWG = NWG, < BG = BG) scenarios for each mycotoxin, calculated for all plant-based alternative products and all seitan products

|  | All plant-based alternative products | | | Seitan products | | |
| --- | --- | --- | --- | --- | --- | --- |
|  | LB | MB | UB | LB | MB | UB |
| DON | 47.0 | 47.4 | 47.9 | 164 | 164 | 164 |
| D3G | 0.00 | 7.05 | 14.1 | 0.00 | 7.05 | 14.1 |
| 3-AcDON | 0.30 | 0.72 | 1.14 | 1.07 | 1.34 | 1.60 |
| 15-AcDON | 0.21 | 0.72 | 1.23 | 0.75 | 1.22 | 1.69 |
| HT-2 | 0.56 | 0.67 | 0.79 | 1.97 | 2.01 | 2.06 |
| T-2 | 0.00 | 0.04 | 0.07 | 0.00 | 0.04 | 0.07 |
| AOH | 0.14 | 0.56 | 0.99 | 0.18 | 0.54 | 0.91 |
| AME | 4.03 | 4.04 | 4.05 | 8.00 | 8.01 | 8.02 |
| TeA | 54.1 | 54.2 | 54.3 | 170 | 170 | 170 |
| AFB1 | 0.00 | 0.01 | 0.02 | 0.00 | 0.01 | 0.02 |
| AFB2 | 0.00 | 0.005 | 0.01 | 0.00 | 0.005 | 0.01 |
| AFG1 | 0.03 | 0.03 | 0.04 | 0.00 | 0.01 | 0.02 |
| AFG2 | 0.00 | 0.14 | 0.28 | 0.00 | 0.14 | 0.28 |
| STC | 0.008 | 0.01 | 0.02 | 0.02 | 0.03 | 0.03 |
| OTA | 0.00 | 0.47 | 0.94 | 0.00 | 0.47 | 0.94 |

Supplementary Table 8: Detailed spiking information for the determination of the recovery

| *Analyte* | Absolute amount [ng] | Internal standard | Absolute amount [ng] |
| --- | --- | --- | --- |
| DON | 15.0  100  300  500 | [^13^C_15_]-DON | 10.0 |
| D3G | 18.5  50.0  100  200 | [^13^C_21_]-D3G | 50.0 |
| 3-AcDON | 15.0  30.0  60.0  150 | [^13^C_17_]-3-AcDON | 15.0 |
| 15-AcDON | 15.0  30.0  60.0  150 |  |  |
| HT-2 | 2.00  4.00  10.0  20.0 | [^13^C_22_]-HT-2 | 10.0 |
| T-2 | 0.50  5.00  20.0  50.0 | [^13^C_4_]-T-2 | 2.00 |
| AOH | 10.0  30.0  70.0  100 | [d_4_]-AOH | 20.0 |
| AME | 0.60  5.00  10.0  20.0 | [d_4_]-AME | 0.20 |
| TeA | 3.00  10.0  20.0  50.0 | [^13^C_6_^15^N]-TeA | 20.0 |
| AFB1 | 0.15  1.00  2.00  5.00 | [^13^C_17_]-AFB1 | 0.30 |
| AFB2 | 0.15  1.00  2.00  5.00 | [^13^C_17_]-AFB2 | 0.30 |
| AFG1 | 0.45  2.00  3.00  5.00 | [^13^C_17_]-AFG1 | 1.00 |
| AFG2 | 0.45  2.00  3.00  5.00 | [^13^C_17_]-AFG2 | 1.00 |
| STC | 0.06  0.18  0.30  1.00 | [^13^C_18_]-STC | 0.30 |
| OTA | 1.50  3.00  7.00  10.0 | [^13^C_20_]-OTA | 2.00 |

Supplementary Table 9: Detailed spiking information for the determination of LODs and LOQs

| *Analyte* | Absolute amount [ng] | Internal standard | Absolute amount [ng] |
| --- | --- | --- | --- |
| DON | 1.50  4.50  10.0  15.0 | [^13^C_15_]-DON | 20.0 |
| D3G | 18.5  50.0  100  200 | [^13^C_21_]-D3G | 50.0 |
| 3-AcDON | 0.70  1.50  4.50  10.0 | [^13^C_17_]-3-AcDON | 7.50 |
| 15-AcDON | 0.70  1.50  4.50  10.0 |  |  |
| HT-2 | 0.20  0.60  1.40  2.00 | [^13^C_22_]-HT-2 | 10.0 |
| T-2 | 0.05  0.15  0.35  0.50 | [^13^C_4_]-T-2 | 2.00 |
| AOH | 1.00  3.00  7.00  10.0 | [d_4_]-AOH | 20.0 |
| AME | 0.04  0.10  0.20  0.40 | [d_4_]-AME | 0.20 |
| TeA | 0.30  0.90  2.10  3.00 | [^13^C_6_^15^N]-TeA | 20.0 |
| AFB1 | 0.02  0.05  0.10  0.15 | [^13^C_17_]-AFB1 | 1.50 |
| AFB2 | 0.02  0.05  0.10  0.15 | [^13^C_17_]-AFB2 | 1.50 |
| AFG1 | 0.02  0.05  0.10  0.15 | [^13^C_17_]-AFG1 | 1.50 |
| AFG2 | 0.15  1.00  2.00  5.00 | [^13^C_17_]-AFG2 | 1.00 |
| STC | 0.003  0.01  0.02  0.06 | [^13^C_18_]-STC | 0.10 |
| OTA | 1.50  3.00  7.00  15.0 | [^13^C_20_]-OTA | 3.00 |

Supplementary Table 10: Detailed spiking information for the determination of precision

| *Analyte* | Absolute amount [ng] | Internal standard | Absolute amount [ng] |
| --- | --- | --- | --- |
| DON | 15.0 | [^13^C_15_]-DON | 20.0 |
| D3G | 50.0 | [^13^C_21_]-D3G | 50.0 |
| 3-AcDON | 4.50 | [^13^C_17_]-3-AcDON | 7.50 |
| 15-AcDON | 4.50 |  |  |
| HT-2 | 2.00 | [^13^C_22_]-HT-2 | 10.0 |
| T-2 | 0.50 | [^13^C_4_]-T-2 | 0.50 |
| AOH | 10.0 | [d_4_]-AOH | 10.0 |
| AME | 0.60 | [d_4_]-AME | 0.20 |
| TeA | 3.00 | [^13^C_6_^15^N]-TeA | 20.0 |
| AFB1 | 0.15 | [^13^C_17_]-AFB1 | 0.30 |
| AFB2 | 0.15 | [^13^C_17_]-AFB2 | 0.30 |
| AFG1 | 0.15 | [^13^C_17_]-AFG1 | 1.00 |
| AFG2 | 2.00 | [^13^C_17_]-AFG2 | 1.00 |
| STC | 0.06 | [^13^C_18_]-STC | 0.30 |
| OTA | 5.00 | [^13^C_20_]-OTA | 3.00 |
